# A fresh pH-responsive imipenem-loaded nanocarrier against *Acinetobacter baumannii* with a synergetic effect

**DOI:** 10.3389/fbioe.2023.1166790

**Published:** 2023-04-11

**Authors:** Shumin Gui, Xisheng Li, Mingming Feng, Hui Liu, Liwenhui Huang, Xinqing Niu

**Affiliations:** ^1^ Department of Hematology, The Third Affiliated Hospital of Xinxiang Medical University, Xinxiang Medical University, Xinxiang, Henan, China; ^2^ Department of Laboratory Medicine, The Third Xiangya Hospital, Cental South University, Changsha, Hunan, China; ^3^ Henan Key Laboratory of Immunology and Targeted Drugs, School of Medical Technology, Xinxiang Medical University, Xinxiang, Henan, China

**Keywords:** *Acinetobacter baumannii*, Imi@ZIF-8, antibacterial infection, synergistic effect, antibiofilm

## Abstract

In recent years, the treatment of *Acinetobacter baumannii* infections has become a pressing clinical challenge due to its increasing incidence and its serious pathogenic risk. The research and development of new antibacterial agents for *A. baumannii* have attracted the attention of the scientific community. Therefore, we have constructed a new pH-responsive antibacterial nano-delivery system (Imi@ZIF-8) for the antibacterial treatment of *A. baumannii*. Due to its pH-sensitive characteristics, the nano-delivery system offers an improved release of the loaded imipenem antibiotic at the acidic infection site. Based on the high loading capacity and positive charge of the modified ZIF-8 nanoparticles, they are excellent carriers and are suitable for imipenem loading. The Imi@ZIF-8 nanosystem features synergistic antibacterial effects, combining ZIF-8 and imipenem to eliminate *A. baumannii* through different antibacterial mechanisms. When the loaded imipenem concentration reaches 20 µg/mL, Imi@ZIF-8 is highly effective against *A. baumannii in vitro*. Imi@ZIF-8 not only inhibits the biofilm formation of *A. baumannii* but also has a potent killing effect. Furthermore, in mice with celiac disease, the Imi@ZIF-8 nanosystem demonstrates excellent therapeutic efficacy against *A. baumannii* at imipenem concentrations of 10 mg/kg, and it can inhibit inflammatory reaction and local leukocyte infiltration. Due to its biocompatibility and biosafety, this nano-delivery system is a promising therapeutic strategy in the clinical treatment of *A. baumannii* infections, providing a new direction for the treatment of antibacterial infections.

## Introduction

*Acinetobacter baumannii* is an aerobic, gram-negative coccobacillus and is a conditional pathogen that can cause community-acquired and hospital-acquired infection (Whiteway et al., 2022). In clinical settings, it is responsible for ventilator-associated pneumonia, catheter-associated blood and urinary tract infections, sepsis, endocarditis, skin and wound infections and meningitis (Chen, 2020). *A. baumannii* is ubiquitous in nature and persistent in the hospital environment, where it often infects immunocompromised patients, particularly those in intensive care units (Shan et al., 2022). According to worldwide data, the detection rate of *A. baumannii* in intensive care units rose from 4% to 7% between 1986 and 2003. Moreover, ICU mortality increased from 53.3% to 84.3% in patients with respiratory-associated pneumonia due to extensively drug-resistant *A. baumannii* (Saipriya et al., 2020). *A. baumannii* was regarded as ESKAPE pathogens. In order to study and develop effective antibacterial drugs against *A. baumannii* with acquired resistance to antibiotics, the WHO has placed it on priority list (Tacconelli et al., 2018). So, there is a need for research into combatting this pathogen.

Genomic and phenotypic identification analyses were performed, demonstrating the association between *A. baumannii* infection and multiple virulence factors, including outer membrane proteins, lipopolysaccharides, capsular polysaccharides, phospholipases, protein secretion systems, quantum sensing and biofilm production (Harding et al., 2018; Dehbanipour and Ghalavand, 2022). These virulence factors contribute to the colonization of pathogenic bacteria and exacerbate antibiotic resistance. At present, antibiotics for the treatment of *A. baumannii* infection are mainly selected based on the sensitivity of pathogens to antibiotics, the severity of the disease and the infection site. Based on its pathogenicity, the clinical treatments of *A. baumannii* include sulbactams, carbapenems, aminoglycosides, polymyxin, tegacycline and combined antibiotic therapy (Fishbain and Peleg, 2010; Isler et al., 2019). Carbapenems are highly effective broad-spectrum antibacterial drugs that exhibit strong antibacterial activity against gram-positive bacteria, gram-negative bacteria and anaerobes and are regarded as the “last line of defense” of antibiotics. Their antibacterial mechanism is similar to β-like lactam antibiotics, as they inhibit the formation of the bacterial cell wall by binding with penicillin-binding proteins (PBPs), leading to inactivation (Papp-Wallace et al., 2011; Skariyachan et al., 2019). Imipenem is the most widely-used carbapenem antibiotic in clinical practice and has high bactericidal activity, fast bactericidal rate and excellent bacterial inhibition against *A. baumannii*, especially for patients with moderate to severe infections and multi-drug resistant bacterial infections. Moreover, imipenem is a time-dependent antibacterial drug with a post-antibiotic effect on bacteria. Imipenem preferentially combines with PBP2, followed by PBP1a and PBP1b, and has a weak affinity for PBP3. This mechanism of action can reduce the release of lipopolysaccharide during bacteriolysis, providing good biological safety (Rodloff et al., 2006; Zhanel et al., 2007; Nowak and Paluchowska, 2016). However, with the wide application of carbapenem antibiotics in recent years, the drug resistance rate of *A. baumannii* to carbapenems has increased year by year (Jiang et al., 2022). Polymyxins are effective for treating *A. baumannii* infection, with low rates of resistance, but are associated with a higher risk of nephrotoxicity (Liu et al., 2021). The overall economic benefit of high-dose sulbactam or combination therapy is poor, and clinical data on the efficacy and resistance of the other antimicrobials mentioned are limited (Betrosian et al., 2008; Chu et al., 2013). Hence, finding new treatment schemes is an urgent problem.

The researcher has extensively studied and formulated treatment plans against bacterial infection and drug resistance, including the development of new antibiotic resistance inhibitors. However, the research of new antibiotics is time-consuming and costly, and the development of antibacterial resistance is significantly faster than the research and development of new antibiotics. Furthermore, drug-resistant inhibitors mainly inhibit the severe evolution of bacteria into drug-resistant bacteria, but the prevalence of drug resistance within a patient or in the population remains a challenge (Baym et al., 2016; Chang et al., 2022). In recent years, nanomaterials have attracted attention due to their physical and chemical properties, such as structural stability, large specific surface area, high porosity, easy surface modification, structural diversity and biocompatibility. They have been applied to biomedical engineering projects, such as biosensors (Singh et al., 2022), drug carriers (Vangijzegem et al., 2019; Huang et al., 2021; Liu et al., 2022), medical implants (Akgöl et al., 2021) and medical imaging (Kalva et al., 2022). The rise of nanomedicine has enabled advances in antimicrobial therapy.

Nanocarriers can selectively transport antibiotics to the site of infection due to their targeting properties, thus improving drug distribution, increasing the effectiveness of antibiotics, reducing drug side effects and overcoming bacterial resistance (Nazli et al., 2022). Many nanomaterials have been used in antimicrobial therapy, such as inorganic metal nanomaterials (gold, silver, copper, zinc, titanium), metal oxide nanoparticles (copper oxide, zinc oxide, titanium oxide, iron oxide), carbon-based nanomaterials (graphene and its derivatives, graphene quantum dots, carbon quantum dots), and organic nanostructures (chitosan, dendrimers, liposomes, micelles, vesicles), etc. (Modi et al., 2022). The antibacterial mechanisms of nanomaterials involve 1) the use of size, surface properties and other unique physicochemical properties to damage important intracellular components and interfere with the normal physiological metabolic processes of bacteria, ultimately leading to bacterial death (Cheng et al., 2022); 2) the use of the enzyme-like activity of nanomaterials, regulating the level of reactive oxygen species (ROS) to exert a strong bactericidal effect by disrupting bacterial biofilms (Godoy-Gallardo et al., 2021); 3) smart response platforms based on nanomaterials, such as pH, enzymes and temperature to enhance antimicrobial activity (Jiang et al., 2020); 4) the use of external stimulus-response properties of nanomaterials, such as light and microwaves, or synergistic antimicrobial drugs to achieve an antimicrobial activity in a single or combined treatment (Díez-Pascual, 2020; Nazli et al., 2022). Zeolite imidazole ester skeleton—8 (ZIF—8) is a porous crystalline material formed by the coordination self-assembly of zinc ion and 2-methylimidazole. ZIF-8 has not only high loading capacity but also has antibacterial activity, acid sensitivity, low cytotoxicity, and good biocompatibility, providing broad applications in biomedicine (Lian et al., 2022). The pH of the microenvironment of infected tissues is slightly lower than that of normal tissues due to acid production at the site of infection, so ZIF-8 can be used as an acid-responsive drug carrier for antimicrobial therapy with superior loading capacity, controlled drug release and enhanced targeting (Abdelhamid, 2021; Tan et al., 2022).

In summary, a novel antimicrobial nanodelivery system with pH acid response function was constructed by synthesizing positively-charged ZIF-8 (PEI@ZIF-8) and loading negatively-charged imipenem onto ZIF-8 nanoparticles (Imi@ZIF-8) using positive and negative charge adsorption forces under ultrasonic stirring. The loaded imipenem is effectively released at the site of bacterial infection due to the acidic microenvironment, displaying the antimicrobial synergy between imipenem and ZIF-8. In addition, subsequent research and experiments found that the Imi@ZIF-8 nanodrug system exerts a strong killing effect on *A. baumannii*. The development of an antibacterial nanosystem with good biocompatibility and biosafety provides a new strategy for the clinical treatment of *A. baumannii*.

## Materials and methods

### Materials

Imipenem was purchased from Aladdin Technology (China). Fetal bovine serum (FBS) and high glucose (Dulbecco’s modified Eagle’s medium DMEM) were obtained from Life Technologies. Dialysis membranes (2000 D), hematoxylin and eosin (HE), crystal violet (CV), and phenazine methosulfate were provided by Solarbio Technology (China). Mueller-Hinton (MH) broth was obtained from Solarbio. Polycarbonate porous membrane syringe filters (200 nm) were obtained from Whatman. The bacterial stock solution and reactive oxygen species (ROS) assay kits were purchased from Beyotime Technology (China). The LIVE/DEAD backlight bacterial viability kit was purchased from Yeasen Biotechnology (China). Anti-IL-6 and anti-Ly6G antibodies were manufactured by BOSTER Biological Technology (China). The HRP-conjugated goat anti-mouse IgG was purchased from BOSTER Biological Technology.

### Experimental cells and experimental animals

The mouse lung epithelial (MLE-12) cell line was provided by the Department of Laboratory Medicine, Xiangya Medicine School, Central South University. MLE-12 cells were cultured in a sterile environment at 37°C and 5% CO_2_. We prepared 5 mL of 10% fetal bovine serum, 45 mL of DMEM (high sugar type) and 1 mL of penicillin-streptomycin (1%) to form the complete medium for the cells used. The 6-week-old female BALB/c mice used in our experiments were purchased from Shrek Laboratory Animals Ltd. in Hunan, China. All mice were kept in specific pathogen-free conditions at the Animal Resource Center of Xinxiang Medical University. All animal experiments were approved by the Xinxiang Medical University Experimental Animal Ethics Committee. The ethical approval code is XYLL-20230027.

### Bacterial strains and bacterial cultures

The *A. baumannii* strains were obtained from the Department of Laboratory Science, The Third Affiliated Hospital of Xinxiang Medical University (Xinxiang, Henan, China). The bacteria were stored in bacterial lyophilization solution at −80°C. A disposable sterile inoculating loop was used to collect the lyophilized solution, and the bacteria were allowed to grow on blood agar plates by triple zoning, followed by incubation at 5% CO_2_ and 37°C for 24 h. Finally, individual colonies from the plates were lysed in a sterile LB broth liquid medium with a disposable sterile inoculating loop and incubated overnight at 200 rpm at 37°C to allow bacterial to growth to their logarithmic phase. The overnight culture was added to a fresh, sterilized LB broth medium, and the OD_600_ value was measured using an enzyme marker at 0.6. All experiments were repeated three times, and the average of the three values was taken.

### Preparation of PEI@ZIF-8

2-methylimidazole was dissolved in methanol to form solution A, and zinc nitrate was dissolved in methanol to form solution B. Solutions A and B were mixed and allowed to react for a certain period of time, and ZIF-8 was obtained by centrifugation (Wang et al., 2020). The precipitate was washed then three times with water. ZIF-8 and PEI were mixed and stirred for a certain period of time, and the PEI-modified ZIF-8 was collected by centrifugation. Finally, the precipitate was washed three times with water.

### Determination of encapsulation and loading rates of imipenem in Imi@ZIF-8

1 mg of PEI@ZIF-8 was dissolved in 1 mL of deionized water (ddH_2_O), and 1.2 mg of imipenem was added and stirred for 4–5 h at room temperature with a magnetic stirrer. The solution was left for 12 h in darkness, then the precipitate was centrifuged in darkness to obtain Imi@ZIF-8. The maximum absorbance of imipenem in the supernatant was determined by UV-visible spectrophotometry (Yu et al., 2014; Wang et al., 2016). The standard concentration gradient of imipenem was set, and the standard curve of concentration and absorbance was established. Then, the content of unbound imipenem in the supernatant was calculated.

The calculation formulas of encapsulation efficiency (EE) and loading efficiency (LE) are as follows:

$$EE=\left(\frac{imipenem\;added-imipenem\;remaining\;in\;supernatant}{imipenem\;added}\right)\times 100\%.$$

$$LE=\left(\frac{amount\;of\;imipenem\;added-amount\;of\;imipenem\;remaining\;in\;the\;supernatant}{total\;weight\;of\;Imi@ZIF-8}\right)\times 100\%.$$

### Characterization of Imi@ZIF-8

Transmission electron microscopy (TEM) was performed to observe the particle size and morphology of ZIF-8, while the surface morphology of ZIF-8 was assessed using Scanning electron microscopy (SEM). A zeta potential-particle size analyzer was used to evaluate imipenem, ZIF-8, and Imi@ZIF-8, whereas dynamic light scattering instrument (DLS) was performed to analyze the hydrated particle size of ZIF-8 and Imi@ZIF-8. The characteristic absorption peak positions of ZIF-8, imipenem and Imi@ZIF-8 were detected by a ultraviolet-visible (UV-vis) spectrophotometer to further validate the successful construction of the nano-delivery system.

### Release characteristics of Imi@ZIF-8

Imipenem release from Imi@ZIF-8 was monitored at pH = 7.4 and pH = 6.5 to investigate the acidic pH response properties of Imi@ZIF-8. Specifically, 5 mL of Imi@ZIF-8 was packed into dialysis bags, which were then immersed in 50 mL of PBS solution at pH = 7.4 and pH = 6.5, respectively (Zhou et al., 2022). The dialysate was collected at 6, 12, 18, and 24 h. The absorbance value of imipenem in the dialysate at 299 nm was measured using a UV-Vis spectrophotometer to determine the cumulative release of imipenem from the solution, and the percentage release—time curves at pH = 7.4 and pH = 6.5 were plotted. All experiments were repeated three times, and the average of the experimental values was taken.

### *In vitro* antibacterial and anti-biofilm effects of Imi@ZIF-8

#### Minimum inhibitory concentration (MIC)

The minimum inhibitory concentration (MIC) of imipenem and ZIF-8 against *A. baumannii* was determined using the broth dilution method. A suspension of *A. baumannii* at a concentration of 1.5 × 10^6^ CFU/mL was added to a 96-well plate. First, the imipenem solution was prepared by serial 2-fold dilution to a final concentration of 0, 0.5, 1, 2, 4, and 8 μg/mL of imipenem in each well and a volume of 100 μL. A suspension of 1.5 × 10^6^ CFU/mL of *A. baumannii* was then prepared, and 100 μL of the suspension was added to each well using a pipette. The suspensions were incubated for 16–20 h at 37°C and 5% CO_2_. Similarly, the minimum inhibitory concentration of ZIF-8 against *A. baumannii* was tested.

#### Disc diffusion method

After obtaining individual colonies, the concentration of *A. baumannii* solution was adjusted to 0.5 MCF with physiological saline, and the solution was applied to MH agar with a sterile cotton swab in a flat layer. After placing a filter paper disc on the staining medium, different contents of the imipenem, ZIF-8 and Imi@ZIF-8 were injected into the paper. The MH agar plates were incubated at 5% CO_2_, 37°C for 24 h, and the size of the inhibition circles was observed and measured.

#### Live/dead backlight bacteria assay

*A. baumannii* was incubated with the corresponding treatment group materials for 24 h, then centrifuged and washed with sterile PBS 1–2 times, the precipitation was the treated *A. baumannii*. The DMAO/EthD—Ⅲ mixed fluorescent dye was prepared according to the manufacturer’s instructions, and the bacteria were resuspended with the prepared fluorescent dye mixture. The two mixtures were mixed and incubated at room temperature for 15–20 min in darkness. Subsequently, 15 µL of the stained bacterial suspension was dropped onto a sterile slide and covered with an 18 mm square sterile slide. The survival of bacteria was observed under a laser confocal microscope (CLSM).

#### Crystal violet staining

100 μL of drug culture solution was added to each well of a 96-well cell culture plate, and 100 μL of an overnight culture of *A. baumannii* was inoculated at 37°C for 24 h to allow the cells to adhere to the wall. PBS was used to carefully wash three times to remove planktonic bacteria, and the bacterial biofilm was fixed with a formaldehyde solution. The samples were left to dry naturally, and 200 μL of 1% crystal violet dye was added to each well and left for 15 min at room temperature. The stain was then washed off 2–3 times with PBS, left to dry, and lysed using 95% ethanol. Then, the solution was incubated in an incubator at 37°C for 25 min. The absorbance value of the lysate was measured using an enzyme marker (570 nm) to determine the biofilm biomass.

The percentage of biofilm eradication and inhibition was calculated as follows:

$$Biofilm\;eradication/inhibition\;rate\;\left(\%\right)=\left(1-\frac{OD\;experiment}{OD\;control}\right)\times 100\%.$$

#### Confocal laser scanning microscope analysis

The biofilms of *A. baumannii* were formed in laser-scanning confocal Petri dishes, and the bacterial biofilms were fluorescently stained with a live/dead bacterial fluorescent dye kit to observe the survival of the biofilms under a CLSM.

### *In vitro* antibacterial mechanism of Imi@ZIF-8

#### Reactive oxygen species (ROS) measurement

PBS, Imipenem, ZIF-8 and Imi@ZIF-8 solutions were added to the bacterial suspension (1 × 10^6^ CFU), co-cultured for 24 h and then resuspended in sterile saline. 1 μL of 2,7-dichlorodihydrofluorescein diacetate (DCFH-DA) fluorescent dye was added in the dark and incubated at 37°C for 1 h. The fluorescence value (excitation/emission wavelength 485/535 nm) was measured with a fluorescence enzyme marker, with the fluorescence intensity of 2,7-dichlorofluorescein (DCF) being proportional to the level of ROS (Scott et al., 1998; Hsieh et al., 2001).

#### Determination of malondialdehyde (MDA)

*A. baumannii* was incubated with each group of drug culture solution for 24 h, centrifuged (12,000 rpm, 2 min), and the supernatant was discarded and resuspended in 1 mL of 2.5% (w/v) trichloroacetic acid. The solution was centrifuged (12,000 rpm, 20 min, 4°C) again to collect the supernatant, and 1 mL of 5% thiobarbituric acid (TBA) solution was added for dilution. An equal volume of 20% (w/v) TCA was added to the mixture; the reaction was carried out in a water bath at 100°C for 30 min, and the mixture was centrifuged (12,000 rpm, 20 min, 4°C). The final absorbance value was measured at 532 nm, and the MDA content (pg/mL) was calculated based on the molar extinction coefficient (1.56 nM^−1^cm^−1^).

#### Construction of the mouse peritonitis model in *Acinetobacter baumannii*

The 6-week-old female BALB/c mice were randomly divided into four groups: the PBS group, imipenem group, ZIF-8 group, and Imi@ZIF-8 group. The mice were infected with an intraperitoneal injection of 150 μL containing 1 × 10^6^ CFU/L *A. baumannii*. After 12–24 h, inflammation, necrosis and infiltration of inflammatory cells (neutrophils, lymphocytes and macrophages) were observed in the liver tissue of the mice, indicating the successful establishment of the abdominal infection mouse model. After the successful construction of the mouse model, the drug was administered once daily, and the injection continued for 3 days. The dosing concentration for each group was calculated based on the imipenem concentration, achieving a final dose of 10 mg/kg per group. After the seventh day of administration, whole blood was collected from the mice, and the liver tissues were stained with hematoxylin and eosin (HE) to observe the inflammatory infiltration and necrosis of liver tissues. Immunohistochemical staining was performed to detect the pro-inflammatory factors interleukin-6 (IL-6) and the neutrophil-specific marker Ly6G.

#### Biocompatibility and biosafety of Imi@ZIF-8

The biocompatibility of Imi@ZIF-8 was assessed using the CCK-8 method. MLE-12 cells (2 × 10^3^/well) were inoculated in a 96-well plate and incubated for 24 h, washed 1–2 times with sterile PBS solution, and ZIF-8 was prepared at a concentration gradient of 10, 20, 40, 60, 80, 100, and 120 μg/mL (using DMEM medium as solvent). After incubation with MLE-12 cells for 24 h, CCK-8 solution (10 µL) was added to each well for 3 h, and the absorbance value was measured at 450 nm. Healthy female BALB/c mice at 6 weeks of age were randomly divided into four groups and injected with PBS, Imipenem, ZIF-8 or Imi@ZIF-8 *via* the tail vein. After 1 week, whole blood was collected, and blood biochemical parameters (RBC, WBC, PLT, CRP, ALT, AST, BUN, and CREA) were measured. Mice were executed by cervical dislocation. The major organs (heart, liver, spleen, lungs and kidneys) were taken for hematoxylin and eosin (HE) staining, and the lesions were observed under the microscope.

### Statistical analysis

All data were expressed as mean ± standard deviation, and statistical analysis was performed using SPSS 20.0 software. Differences between groups were analyzed using oneway ANOVA, followed by further analysis using Tukey’s post-test (*p < 0.05, **p < 0.01, ***p < 0.001).

## Results and discussion

### Construction and characterization of the Imi@ZIF-8 nano-drug delivery system

**FIGURE 1:**
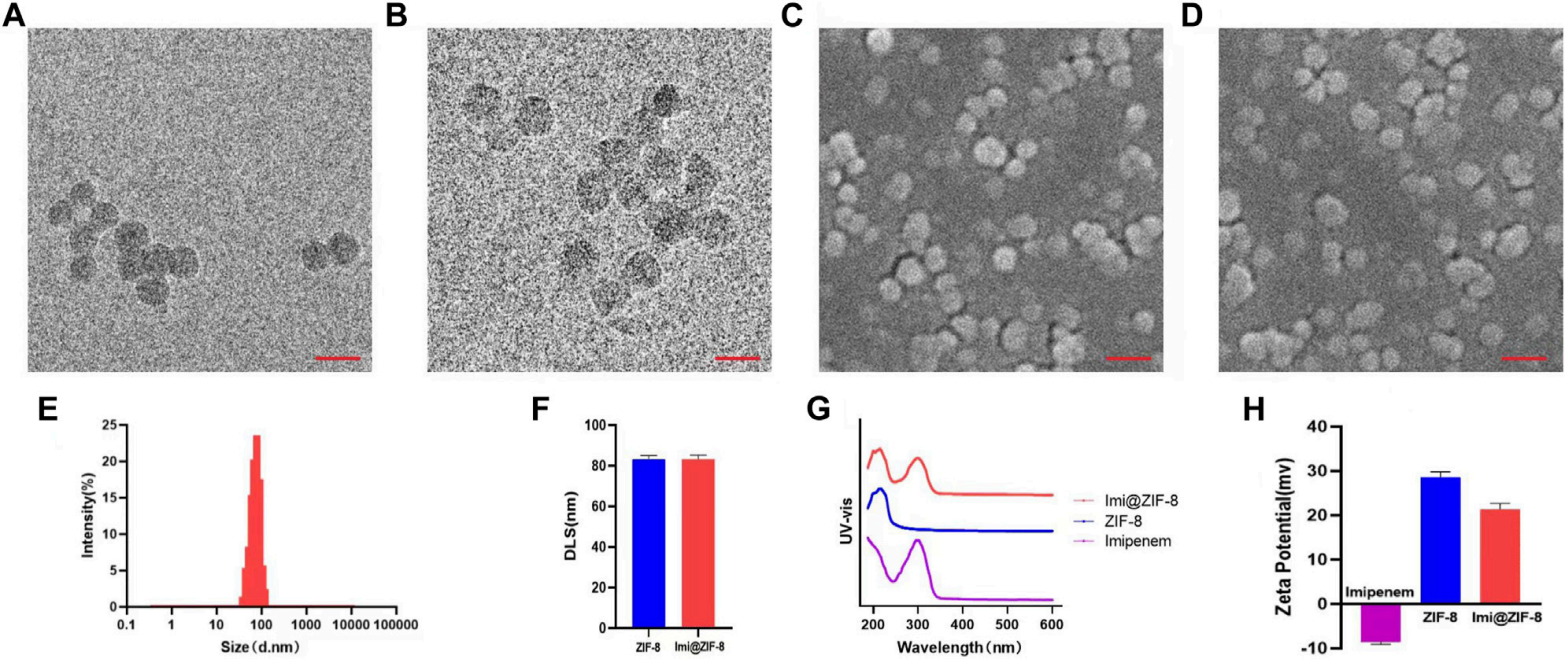
Characterization of ZIF-8 and Imi@ZIF-8. **(A)** TEM image of ZIF-8. **(B)** TEM image of Imi@ZIF-8. Scale bar is 150 nm. **(C)** SEM image of ZIF-8. **(D)** SEM image of Imi@ZIF-8. Scale bar is 200 nm. **(E)** Particle sizes of Imi@ZIF-8 detected by TEM. **(F)** Dynamic light scattering (DLS) analyze the hydrated particle size of ZIF-8 and Imi@ZIF-8. **(G)** UV-vis spectrometries of imipenem, ZIF-8 and Imi@ZIF-8. **(H)** Zeta Potential values of imipenem, ZIF-8 and Imi@ZIF-8. Data are presented as means ± SD (*n* = 3).

In this study, the new antibacterial nano drug delivery system Imi@ZIF-8 was designed and synthesized. The particle size, morphology and dispersion of the prepared nano-drug delivery system were studied by TEM. The positively-charged ZIF-8 synthesized in this experiment was spherical, with an average particle size of about 80 nm and uniform distribution (Figure 1A). The overall morphology of the imipenem-loaded Imi@ZIF-8 showed no significant change, with a spherical shape and a particle size of around 80 nm (Figures 1B, E). SEM confirmed the TEM results, showing both ZIF-8 and Imi@ZIF-8 with particle sizes of around 80 nm and good dispersion (Figures 1C, D). DLS allows analysis of the hydrated particle size of the nanoparticles, complementing the TEM results on nanoparticle size. As shown in Figure 1F, the average hydrated particle size of ZIF-8 and Imi@ZIF-8 was about 80 nm. Moreover, the ζ-potential analysis of imipenem, ZIF-8, and Imi@ZIF-8 was performed separately, as displayed in Figure 1H. The ζ-potential of ZIF-8 after PEI modification showed a positive charge of 28.59 ± 1.39 mV, while imipenem was negatively charged (−8.57 ± 0.59 mV). The zeta potential of Imi@ZIF-8 after loading imipenem was 21.35 ± 1.35 mV. This lower value indicated that ZIF-8 was successfully loaded with imipenem and that the Imi@ZIF-8 nano-delivery system had good stability and dispersibility. Subsequently, UV-vis spectroscopy was performed to verify the encapsulation of imipenem in the ZIF-8 nanoparticles. As illustrated in Figure 1G, ZIF-8 showed a characteristic absorption peak at 216.5 nm, while imipenem had a characteristic absorption peak at 299 nm. Imi@ZIF-8 exhibited both characteristic absorption peaks in UV-vis, corresponding to imipenem (299 nm) and ZIF-8 (216.5 nm), indicating that ZIF-8 was successfully loaded with imipenem. These results provide strong evidence for the successful preparation of the Imi@ZIF-8 nanodrug delivery system.

### *In vitro* release study of the Imi@ZIF-8 nano drug delivery system

**FIGURE 2:**
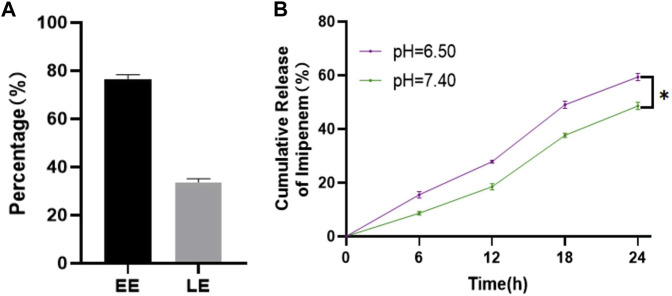
Drug loading and release rate of Imi@ZIF-8. **(A)** EE and LE of Imi@ZIF-8. **(B)** Cumulative release rates of imipenem from Imi@ZIF-8 at different pH values (6.5 and 7.4). Data are presented as means ± SD (*n* = 3). Intergroup comparisons: *p < 0.05.

ZIF-8 is an excellent nano-delivery vehicle due to its large specific surface area and porous structure, and the ZIF-8 nanoparticles were further modified to be positively charged, improving imipenem release. EE and LE of imipenem were determined using a UV-vis spectrophotometer at 299 nm. As shown in Figure 2A, the EE and LE of imipenem in the nano-delivery system were 76.38% ± 2.05% and 33.68% ± 1.53%, respectively. To investigate the pH response of the drug delivery system, the release of imipenem from Imi@ZIF-8 in neutral (pH = 7.4) and acidic environments (pH = 6.5) were evaluated at different time periods. Due to the anaerobic fermentation of bacteria at the site of infection, large amounts of organic acids are produced, creating an acidic microenvironment (Qiao et al., 2019). In this experiment, pH = 6.5 was used to simulate the acidic microenvironment of bacterial infection *in vivo*. The 24 h cumulative release efficiency of imipenem was 48.58% ± 1.49% at pH 7.4% and 59.36% ± 1.39% at pH 6.5 (Figure 2B), indicating that the acidic microenvironment resulted in a higher imipenem release rate. The facilitated release of imipenem from the nano-delivery system at the site of bacterial infection leads to superior antibacterial activity. This facilitated release is due to the protonation of the 2-methylimidazole ligand in ZIF-8 under acidic conditions, resulting in the breaking of the ligand bond between zinc and 2-methylimidazole, releasing imipenem from the nano-loaded system (Zheng et al., 2016; Soomro et al., 2019). The above experimental results indicated that ZIF-8 is a good drug delivery vehicle, and Imi@ZIF-8 is responsive to acidic microenvironments, providing superior antibacterial drug release at the site of bacterial infection.

### *In vitro* antibacterial and anti-biofilm effects of Imi@ZIF-8

**FIGURE 3:**
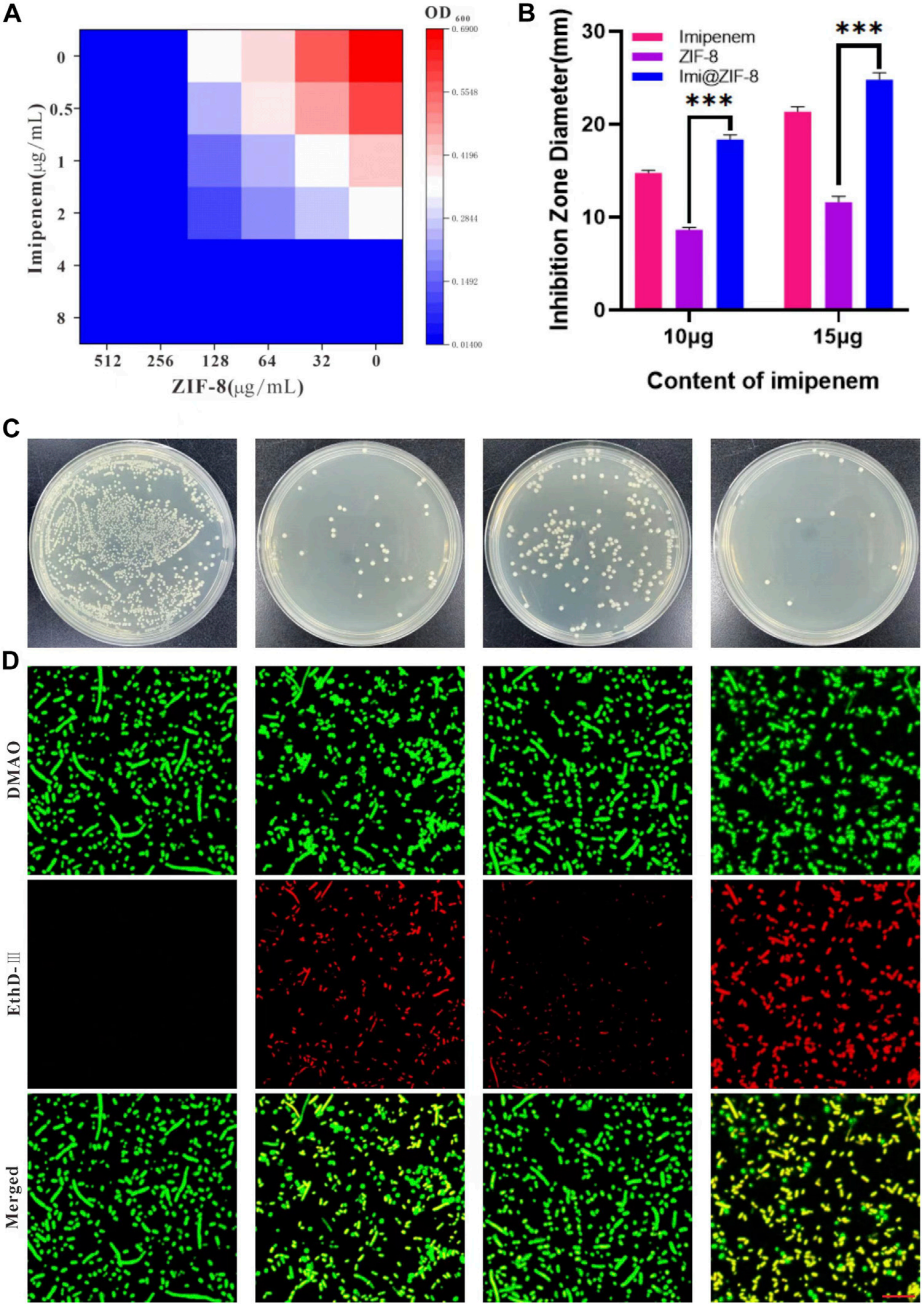
*In vitro* antibacterial effect of Imi@ZIF-8. **(A)** Different concentration of Imipenem and ZIF-8 inhibited on the growth of *A. baumannii*. **(B)** Corresponding inhibition zone diameters of imipenem, ZIF-8 and Imi@ZIF-8 against *A. baumannii*. **(C)** Photographs of agar plates showing the antibacterial activity for PBS, imipenem, ZIF-8 and Imi@ZIF-8 against *A. baumannii*. **(D)** CLSM imaging of death/live staining after *A. baumannii* exposure to various treatments (PBS, imipenem, ZIF-8 and Imi@ZIF-8) for 24 h. Scale bar is 20 μm. Data are presented as mean ± SD (*n* = 3). Compared to ZIF-8: ***p < 0.001.

MIC is a measure of the antimicrobial performance of an antimicrobial agent. A broth dilution method was used to determine the MIC value of the free drugs. As shown in Figure 3A, the MIC value of imipenem and ZIF-8 against *A. baumannii* was 4 μg/mL and 256 μg/mL, respectively. Based on the results, the fractional inhibitory concentration (FIC) index of imipenem and ZIF-8 against *A. baumannii* was calculated to be less than 0.5, which illustrates the synergistic antibacterial effect of imipenem and ZIF-8 against *A. baumannii*. Furthermore, the inhibition activity of free imipenem, ZIF-8 and Imi@ZIF-8 were compared using the disc diffusion method, showing that Imi@ZIF-8 had the largest inhibition circle diameter at similar levels. The diameters of the inhibition circles of 15 μg Imi@ZIF-8, Imipenem and ZIF-8 were 24.81 ± 0.73 mm, 21.39 ± 0.51 mm and 11.64 ± 0.63 mm, respectively (Figure 3B). Figure 3C shows the bacterial counts of *A. baumannii* after 24 h treatment with PBS, ZIF-8, imipenem, and Imi@ZIF-8. A lower number of colonies was observed in the ZIF-8 group compared to the PBS group, but the inhibition effect was not significant. In contrast, the bacterial growth in the free imipenem and Imi@ZIF-8 groups was significantly inhibited, with the Imi@ZIF-8 group showing the most obvious effect. The above results indicate that ZIF-8 can enhance the antibacterial ability of imipenem against *A. baumannii*. Subsequently, the DMAO/EthD-III live/dead bacterial staining kit was used to further observe the bacterial survival status of the free drug and Imi@ZIF-8 groups. Each treatment group underwent live-dead fluorescence staining and was observed under a CLSM. The DMAO fluorescent dye stains dead and alive bacteria green, whereas EthD-III only stains dead bacteria with a red fluorescent dye. The PBS group showed basically no red fluorescence signal, indicating that *A. baumannii* was alive (Figure 3D). However, the ZIF-8, Imipenem, and Imi@ZIF-8 treated groups demonstrated an increasing intensity of red fluorescence, indicating increasing *A. baumannii* death. After 24 h of Imi@ZIF-8 treatment, nearly all *A. baumannii* were dead. In summary, ZIF-8 and imipenem exerted synergistic antibacterial effects against *A. baumannii*, with the Imi@ZIF-8 nano-delivery system being more effective than free imipenem and ZIF-8. Therefore, the nano-delivery system was effective against *A. baumannii* infection.

**FIGURE 4:**
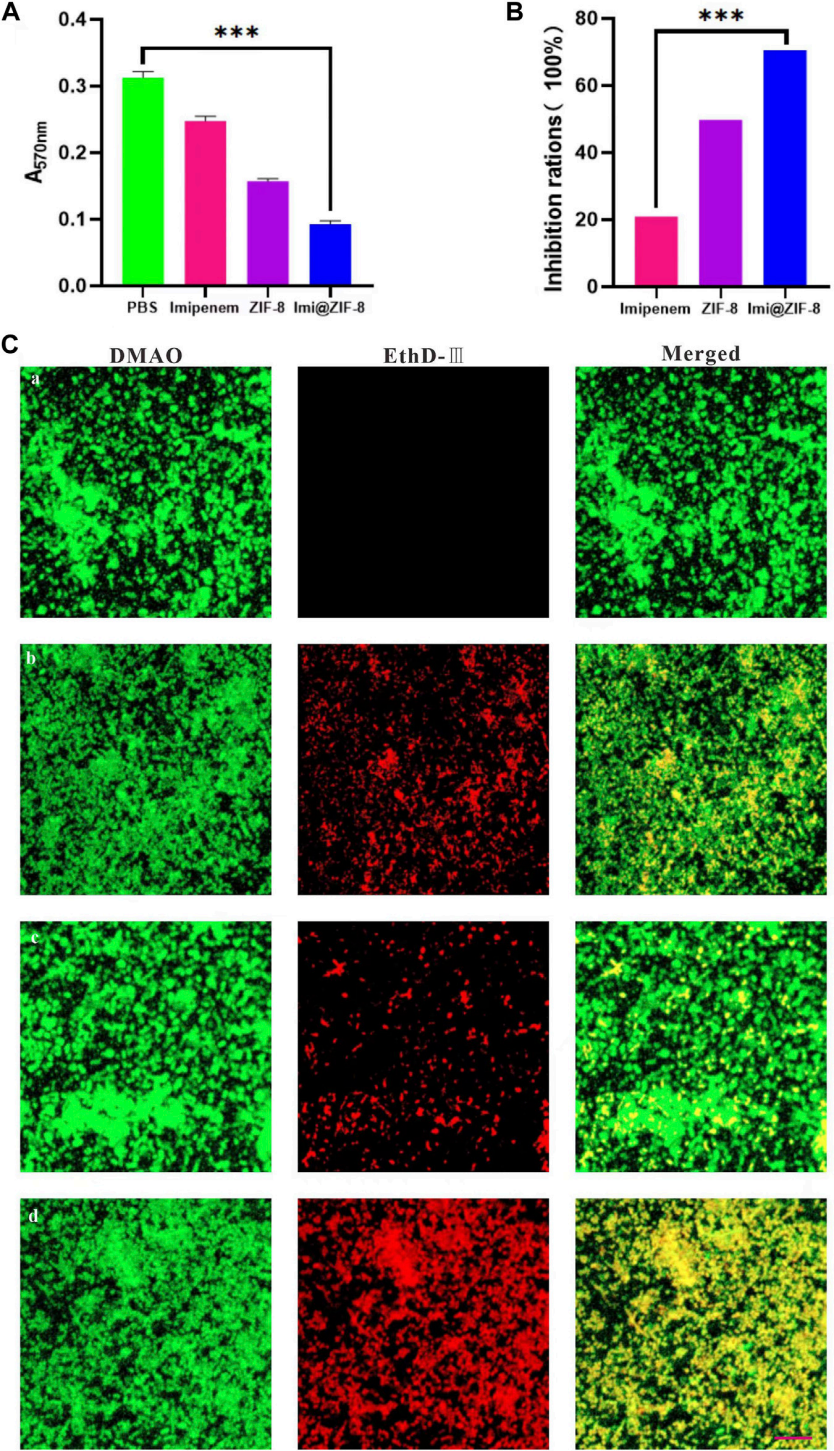
Influence of Imi@ZIF-8 on the biofilm formation and damage of *A. baumannii*. **(A)** CV staining analysis after receiving different treatments for 24 h. The absorbance value was detected using an enzyme marker (570 nm) to reflect biofilm formation. **(B)** CV staining analysis. Relative percentage of biofilm inhibition. **(C)** CLSM images of *A. baumannii* biofilms treated with various treatments (PBS, imipenem, ZIF-8, or Imi@ZIF-8) for 24 h. Scale bar is 20 μm. Data are indicated as mean ± SD (*n* = 3). Compared to the PBS group: ***p < 0.001; Compared to the imipenem group: ***p < 0.001.

Bacterial biofilms are associated with bacterial drug resistance, and the ability to inhibit biofilm formation is an important indicator in determining the clinical application of antimicrobial agents (Sharahi et al., 2019). Crystalline violet staining (CV) is a commonly used assay for early and mid-stage biofilm formation, with higher absorbance values indicating higher amounts of biofilm production by bacteria. As shown in Figure 4A, the absorbance value of the PBS group at 570 nm after 24 h of treatment is 0.313 ± 0.0097, while the Imi@ZIF-8 group had an absorbance value of only 0.092 ± 0.006; Figure 4B shows the percentage of biofilm inhibition of *A. baumannii* by each treatment group after 24 h of treatment. The Imi@ZIF-8 group demonstrated a biofilm inhibition rate of about 71%, much higher than that of the imipenem group (20.93%) and the ZIF-8 group (49.74%), indicating that Imi@ZIF-8 exerted the strongest inhibitory effect on *A. baumannii* biofilm formation. To investigate the killing ability of the Imi@ZIF-8 nano-delivery system on *A. baumannii* biofilms, the “Live/Dead Bacterial Staining Kit” was used, and the survival of the biofilms was assessed under CLSM. The strongest red fluorescence was observed in the Imi@ZIF-8 group, indicating a basically all-dead biofilm, showing a significantly stronger killing effect on *A. baumannii* than the PBS, imipenem and ZIF-8 groups (Figure 4C).

### Antimicrobial mechanism of Imi@ZIF-8

**FIGURE 5:**
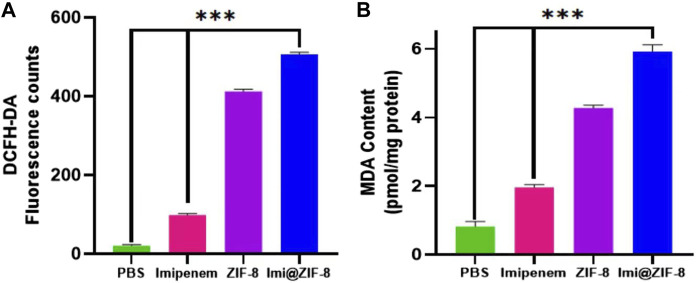
Antibacterial mechanism of Imi@ZIF-8. **(A)** The fluorescence intensity of *A. baumannii* was measured using a fluorometer to reflect ROS formation. **(B)** MDA contents of *A. baumannii* treated with respective materials (PBS, imipenem, ZIF-8 or Imi@ZIF-8) for 24 h represents the extent of membrane damage. Data are presented as mean ± SD (*n* = 3). Compared to the PBS group and the imipenem group: ***p < 0.001.

Although small amounts of ROS are important for maintaining the life cycle of cells, excessive ROS can lead to oxidative stress, disrupting the integrity of bacterial cell membranes and interfering with a range of normal physiological activities, ultimately inducing bacterial death (Hui et al., 2020). Therefore, a DCFH-DA fluorescent probe was used to detect intracellular ROS production in *A. baumannii*. As shown in Figure 5A, the four groups, PBS, Imipenem, ZIF-8 and Imi@ZIF-8, showed increasing ROS production by *A. baumannii*, with the Imi@ZIF-8 group inducing a significantly higher amount of ROS production than the other treatment groups. In addition, nanomaterials may trigger lipid oxidation reactions in bacteria, and the MDA method was used to detect the lipid oxidation levels in *A. baumannii* to reflect the extent of bacterial damage. A significant difference in MDA content was observed between the different treatment groups (Figure 5B), with the highest MDA content produced by *A. baumannii* after Imi@ZIF-8 treatment. These findings suggested that Imi@ZIF-8 induced the most severe damage to *A. baumannii*.

The above results indicated that the Imi@ZIF-8 nano-delivery system effectively eliminated *A. baumannii* by interfering with the normal physiological activities of bacterial cells, catalyzing the production of ROS, inducing lipid oxidation and other antibacterial mechanisms.

### *In vivo* anti-infective effect of Imi@ZIF-8

**FIGURE 6:**
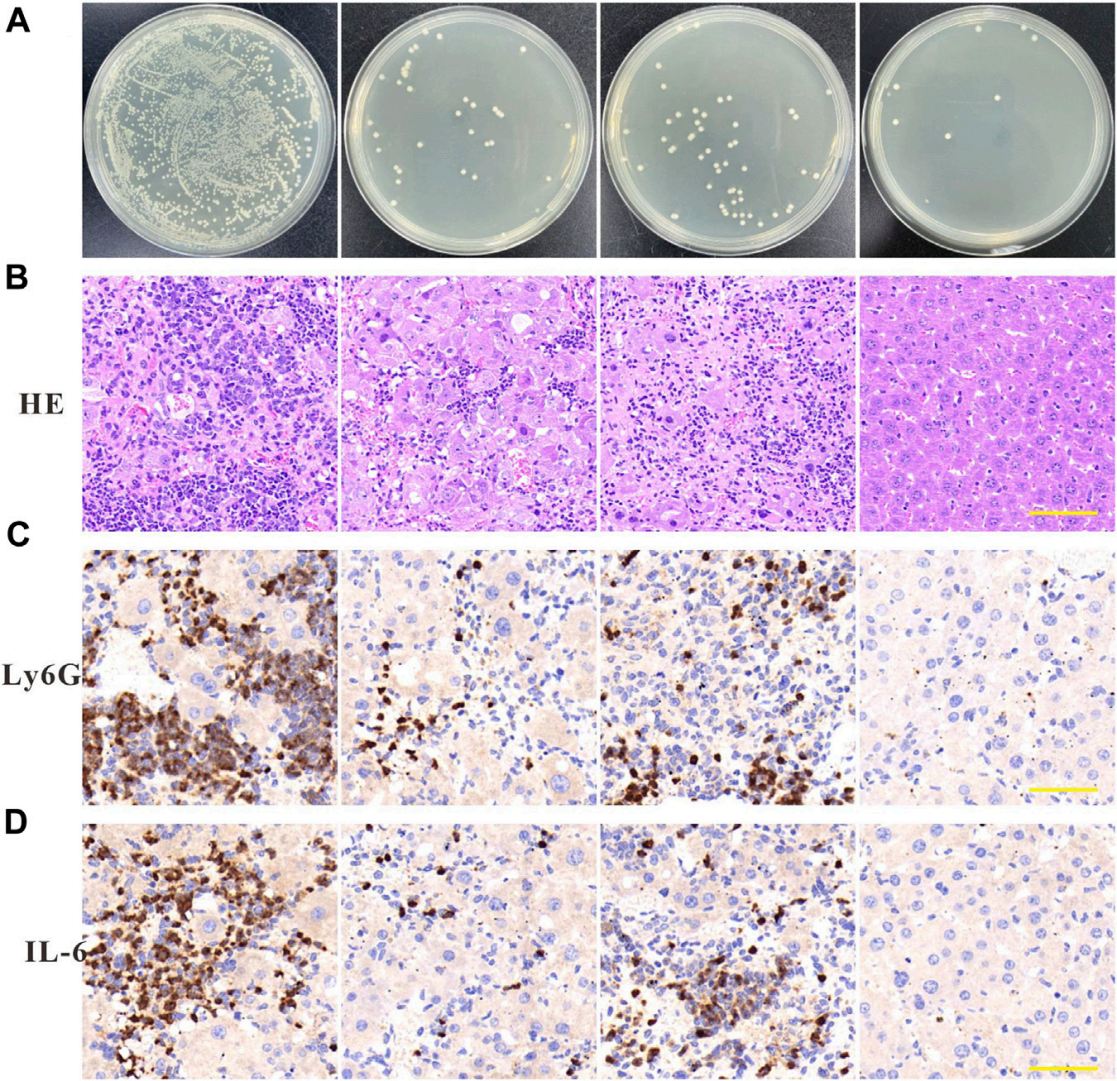
Anti-infection effects of Imi@ZIF-8 *in vivo*. After successful establishment of the mouse peritonitis model in *A. baumannii*, BALB/c mice were intraperitoneal injected with PBS, imipenem, ZIF-8, or Imi@ZIF-8 (10 mg imi/kg). **(A)** Bacterial counting of liver tissues after treated with the respective materials. Data are indicated as means ± SD (*n* = 3). **(B)** Representative images of liver tissues after H&E staining at 7 days after intraperitoneal injection of different treatments. Scale bar is 100 μm. **(C)** Immunohistochemistry staining of Ly6G in liver tissues at 7 days after intraperitoneal injected with PBS, imipenem, ZIF-8, or Imi@ZIF-8, respectively. **(D)** Immunohistochemistry staining of IL-6 in liver tissues. Scale bar is 100 μm.

To further investigate the *in vivo* anti-amastigotes effect of Imi@ZIF-8, a celiac disease mouse model was constructed by infection with *A. baumannii*. The mice with peritonitis were randomly divided into the PBS group, imipenem group, ZIF-8 group and Imi@ZIF-8 treatment groups. After 3 days of continuous tail vein administration, the mice were removed and executed, and the liver tissues were collected under aseptic conditions for bacterial counting. The different treatment groups showed significantly different results. Compared with the other treatment groups, the Imi@ZIF-8 group had the lowest number of *A. baumannii* in the liver tissues, followed by the imipenem group, the ZIF-8 group, and the PBS group (Figure 6A). The pathological hepatic changes in mice with peritonitis were observed by HE staining. Furthermore, the HE section results also provided good evidence of the *in vivo* antibacterial effect of Imi@ZIF-8. As shown in Figure 6B, severe inflammatory cell infiltration and congestion were visible in the liver tissue sections of the PBS group, demonstrating a significant inflammatory response. However, the liver tissue in the Imi@ZIF-8 group was significantly milder than the other treatment groups, with no significant inflammatory cell infiltration and mostly normal liver morphology. Among all the treatments, Imi@ZIF-8 was the most effective in combating *A. baumannii* infection. Subsequently, immunohistochemical staining was used to detect inflammation in the liver tissue following *A. baumannii* infection by determining the expression levels of IL-6 and Ly6G, which was positive when brownish-yellow particles were present. The immunohistochemical images revealed that the increased expression of IL-6 and Ly6G in the imipenem, ZIF-8 and PBS groups, in order, which were significantly higher than those in Imi@ZIF-8 group (Figures 6C, D). These results indicated that Imi@ZIF-8 effectively inhibited the infiltration and inflammatory response of leukocytes and had excellent therapeutic efficacy in mice with celiac disease caused by *A. baumannii*. Collectively, the above *in vivo* and *in vitro* results demonstrated that Imi@ZIF-8 could be an effective novel nano-delivery system against *A. baumannii*.

### Biocompatibility and biosafety of Imi@ZIF-8

**FIGURE 7:**
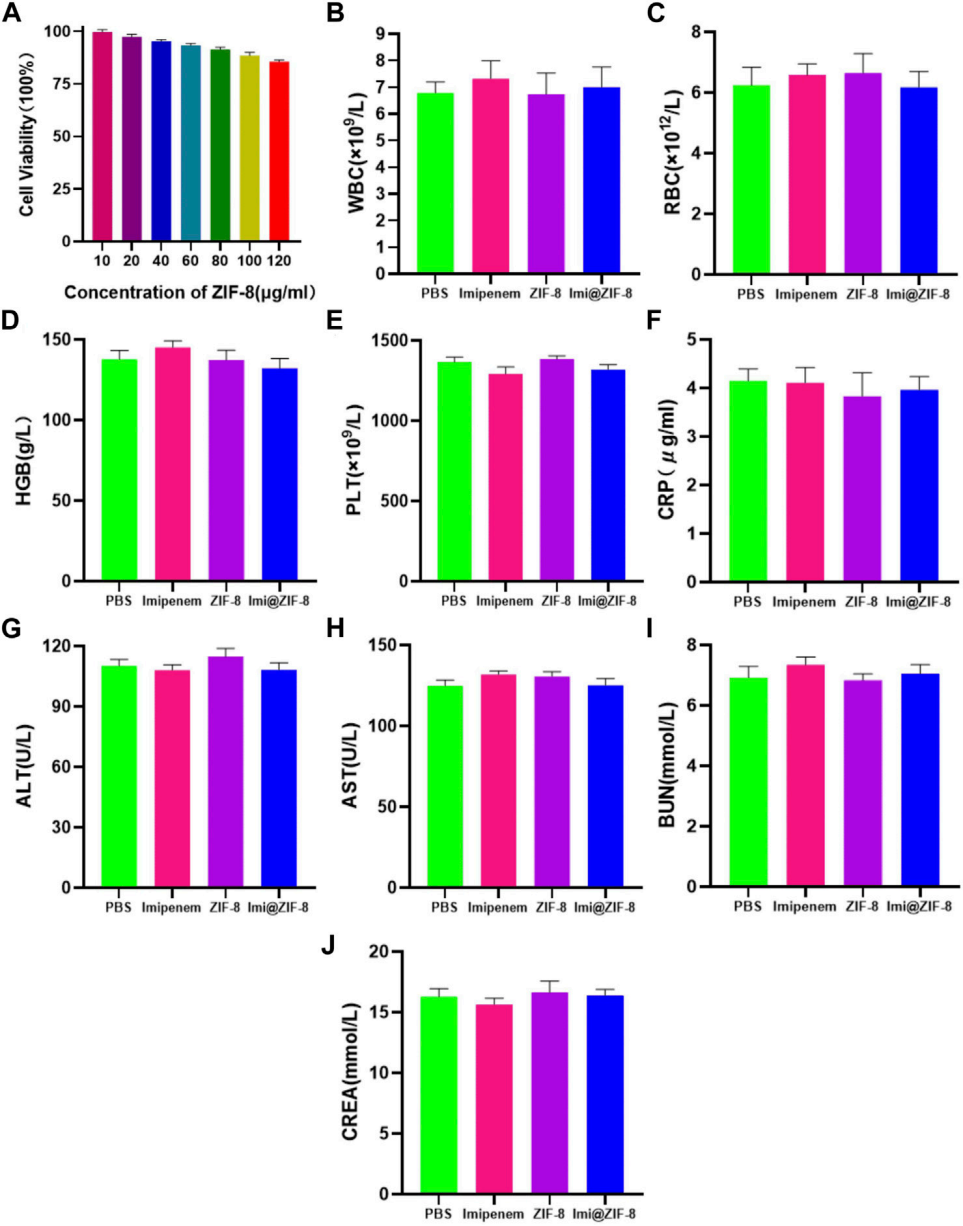
The toxicity assessment of Imi@ZIF-8. **(A)** MLE-12 cells was treated with various concentration of ZIF-8 for 24 h; the cell viability (%) was assayed by CCK-8. **(B–J)** Blood routine and blood biochemical indices of mice at 7 days after intraperitoneal injection of PBS, imipenem, ZIF-8 and Imi@ZIF-8. Data are indicated as means ± SD (*n* = 3).

**FIGURE 8:**
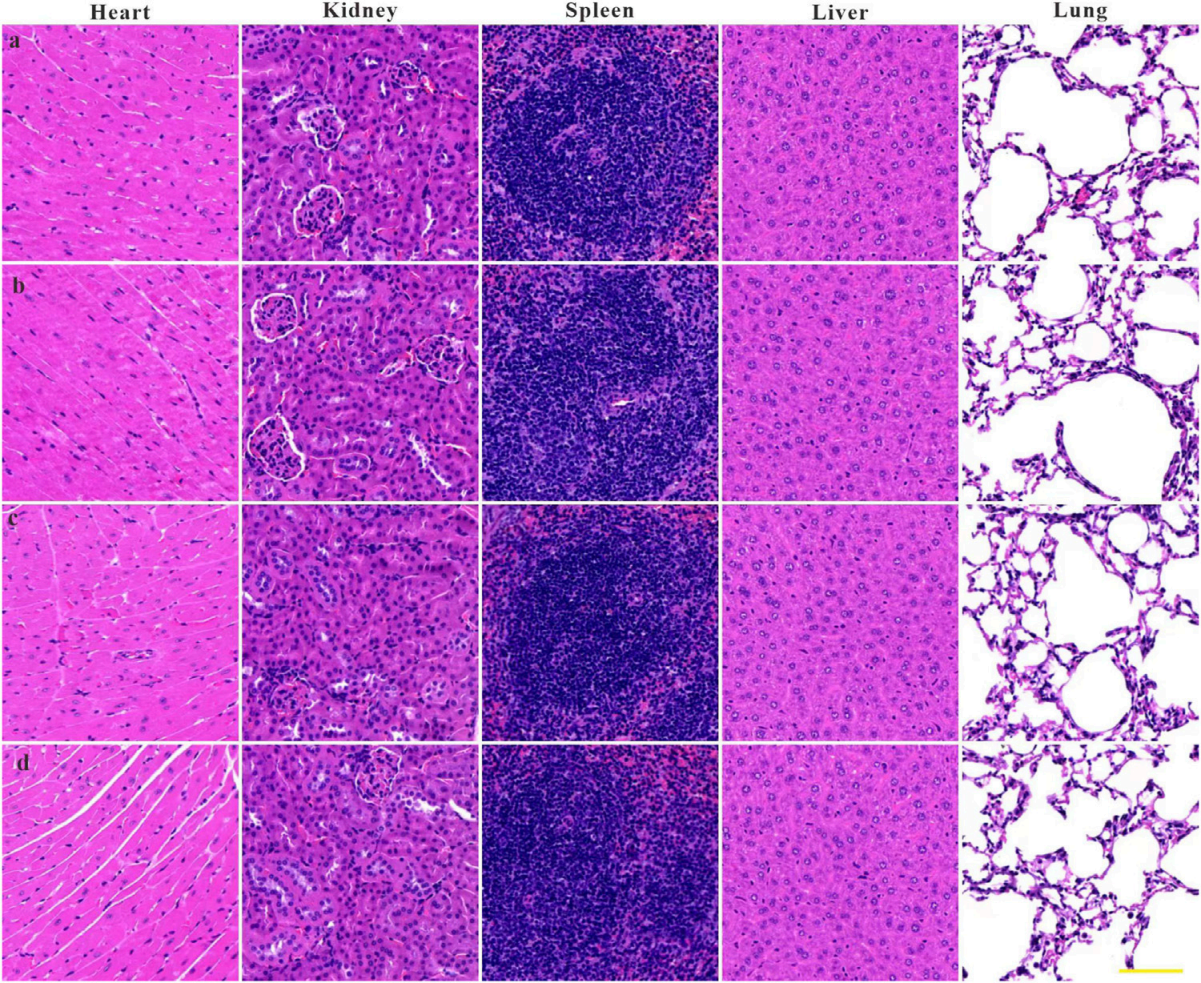
Histological evaluation of heart, liver, spleen, lung, and kidney samples from mice at 7 days upon administration of **(A)** PBS, **(B)** imipenem, **(C)** ZIF-8 and **(D)** Imi@ZIF-8. Scale bar is 100 μm.

To investigate the feasibility of ZIF-8 as a nanodrug carrier for antimicrobial therapy, the biocompatibility and biosafety of the Imi@ZIF-8 nanodrug delivery system were analyzed. MLE-12 cells were treated with different concentrations (10, 20, 40, 60, 80, 100, and 120 μg/mL) of ZIF-8 for 24 h, and the cell viability of the MLE-12 cells was assayed using the CCK-8 method. The results are shown in Figure 7A. MLE-12 cell activity decreased as the concentration of ZIF-8 was increased, but its cell survival rate remained higher than 80%, indicating the good biocompatibility of Imi@ZIF-8. These results have laid a foundation for clinical antibacterial treatment. To assess the *in vivo* safety and toxic effects of ZIF-8 and Imi@ZIF-8, female BALA/c mice were randomly divided into four groups and injected with PBS, Imipenem, ZIF-8, and Imi@ZIF-8, respectively. Hematological parameters were measured to detect the toxicity of the nanomaterials in mice 1 week after tail vein administration, including whole blood parameters such as white blood cells (WBC), red blood cells (RBC), hemoglobin (HGB), and platelets (PLT), C-reactive protein (CRP), liver function parameters alanine aminotransferase (ALT) and glutathione aminotransferase (AST), and renal function parameters such as blood urea nitrogen (BUN), and creatinine (CREA). As shown in Figures 7B–J, no significant difference was observed in all hematological indicators between the groups, indicating no significant toxic effect from ZIF-8 and Imi@ZIF-8. In addition, the pathological changes in the major organs (heart, liver, spleen, lung and kidney) were assessed by HE staining to further clarify the *in vivo* toxicity of ZIF-8 and Imi@ZIF-8, as shown in Figure 8, no inflammation, fibrosis, necrosis or histological abnormalities were observed. The results suggest that ZIF-8 and Imi@ZIF-8 induce no significant damage to the heart, liver, spleen, lungs and kidneys and that they have good biosafety and low toxic side effects. In conclusion, the Imi@ZIF-8 nano-delivery system has good biocompatibility and biosafety.

## Conclusion

The above experimental studies demonstrated the promising potential of our novel antimicrobial nano-delivery system (Imi@ZIF-8) in the treatment of *A. baumannii* infections, showing good biocompatibility, high biosafety, pH acid responsiveness and efficient antimicrobial action. Due to its response to acidic pH, the Imi@ZIF-8 nano-delivery system features an improved release of the loaded imipenem at the acidic bacterial infection sites. Therefore, a synergistic antibacterial effect is achieved between the ZIF-8 and imipenem in the nano-delivery system, further enhancing its antibacterial activity against *A. baumannii*. When the loaded imipenem concentration reaches 20 µg/mL, Imi@ZIF-8 is highly effective against *A. baumannii in vitro*. Moreover, Imi@ZIF-8 exerts excellent therapeutic efficacy against *A. baumannii* at imipenem concentrations of 10 mg/kg and facilitates the inhibition of inflammatory response and leukocyte infiltration in mice with peritonitis, which promotes tissue recovery after bacterial infection. Overall, the Imi@ZIF-8 nano-delivery system provides a new clinical treatment strategy for the antimicrobial treatment of *A. baumannii* and provides a strong foundation for nano-antimicrobial therapy. More studies on the preventive effect of this nanomaterial on bacterial infections can be conducted in the future.

## Data Availability

The original contributions presented in the study are included in the article/Supplementary Material, further inquiries can be directed to the corresponding author.
